# Trans-arterial gemcitabine micro perfusion of locally advanced pancreatic cancer enabled by coil plus glue embolization of a pancreaticoduodenal branch

**DOI:** 10.1016/j.radcr.2026.04.043

**Published:** 2026-05-07

**Authors:** Bela Kis, Olivia Bishop, Elias Salloum, Mustafa Al-Roubaie, Dae Won Kim, Ramtin Agah

**Affiliations:** a Diagnostic Imaging and Interventional Radiology, Moffitt Cancer Center, Tampa, FL, United States; b Gastrointestinal Oncology, Moffitt Cancer Center, Tampa, FL, United States; c RenovoRx, Inc., Los Altos, CA, United States

**Keywords:** Trans-arterial micro perfusion, Chemoperfusion, Arterial embolization, Locally advanced pancreatic cancer

## Abstract

Trans-arterial micro perfusion (TAMP) is a technique that utilizes a novel double-balloon occlusion catheter (RenovoCath) for localized drug delivery in the absence of tumor feeder vessels. Side branch exclusion is a prerequisite for achieving the intra-arterial pressure that drives TAMP. We report a case in which coil plus glue embolization of a vessel side branch successfully enabled TAMP-mediated drug delivery. An 82-year-old male with locally advanced pancreatic cancer was referred for gemcitabine treatment via TAMP. Angiography identified the superior mesenteric artery (SMA) as the best target; however, a pancreaticoduodenal artery (PDA) branch of the SMA could not be excluded. Complete occlusion of the PDA branch was achieved with coil plus glue embolization; TAMP-mediated gemcitabine delivery was then successfully performed in the same setting. In cases where arterial anatomy may otherwise preclude TAMP, coil plus glue embolization of side branches may provide a viable solution.

## Introduction

Trans-arterial micro perfusion (TAMP) is a new technique that employs a novel double-balloon endovascular catheter with an adjustable occlusion distance (RenovoCath, RenovoRx Inc., Mountain View, CA) for targeted delivery of chemotherapeutics in the absence of tumor feeder vessels. RenovoCath’s slidable balloons allow for positioning with arterial side branch exclusion, thereby precluding therapeutic washout while driving the infusate across the vessel wall via increased intra-arterial pressure [1]. Early data from an ongoing Phase 3 trial (NCT03257033) show a significant reduction in systemic side effects with TAMP-mediated gemcitabine delivery versus systemic delivery in patients with locally advanced pancreatic cancer (LAPC) [2]. However, despite RenovoCath’s adjustability, side branch exclusion—which is a pre-requisite for achieving pressure mediated TAMP—can still be difficult with challenging arterial anatomy (ie, multiple jejunal branches off the proximal superior mesenteric artery [SMA]). Herein, we present such a case in which TAMP was successfully enabled by coil plus glue embolization of a pancreaticoduodenal artery (PDA) branch of the SMA. Institutional review board approval was not required for this case report.

## Case presentation

The patient is an 82-year-old male, with previous medical history of *Helicobacter pylori* infection, basal cell cancer, chronic pancreatitis and colon diverticular disease. His family history was significant for colon cancer of his mother and prostate cancer of his father. In May 2024 he presented with epigastric pain and 5 kg unintentional weight loss. Non-contrast CT scan demonstrated stigmata of chronic pancreatitis of parenchymal atrophy with punctate calcification, CT scan also revealed ductal dilatation in the pancreatic body and tail, but no obvious mass lesion was seen. Follow-up MRI revealed a 2.3 × 2.2 cm mass in the pancreatic neck encasing the SMA which showed restricted diffusion and was hypoenhancing on postcontrast sequences. There was abrupt moderate upstream segmental dilatation of the main pancreatic duct involving the pancreatic body and tail. He underwent upper gastrointestinal endoscopy with ultrasound and fine needle aspiration of the pancreatic mass. The cytology showed adenocarcinoma. Due to vascular involvement of the tumor he was diagnosed with LAPC, and was not a candidate for resection and was started on systemic gemcitabine/paclitaxel chemotherapy. In January 2025, the patient underwent stereotactic body radiotherapy and was then referred for intra-arterial gemcitabine infusion via TAMP. His relevant lab values were the following: hemoglobin 9.2 g/dl, white blood count 6400/ µL, platelet count 145,000/µL, INR 1.1, creatinine 0.8 mg/dl, albumin 3.6 g/dl, bilirubin 0.5 mg/dl, Ca-19-9 26.5 U/mL (normal < 37 U/mL).

**Fig. 1:**
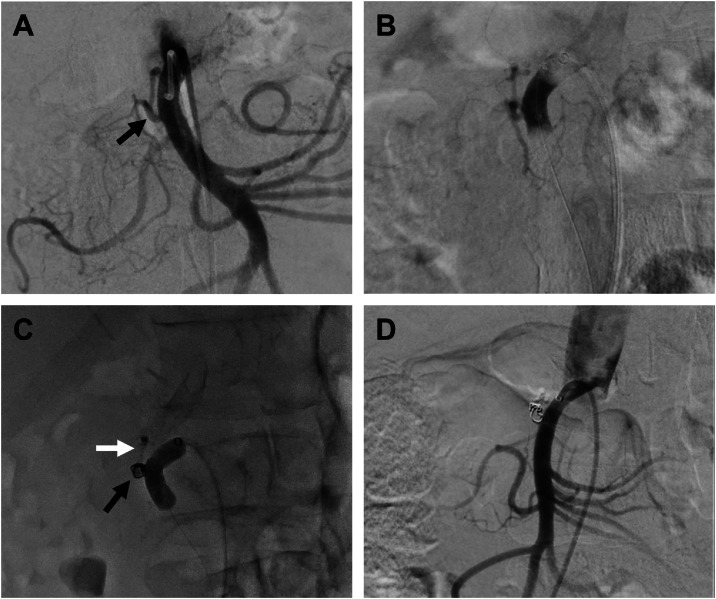
Angiograms of superior mesenteric artery (SMA). A–C: Day of initial procedure. (A) Digital subtraction angiogram (DSA) of the SMA. Black arrow points to the inferior pancreaticoduodenal artery (PDA). (B) Double balloon exclusion of the proximal SMA showing contrast opacification of the inferior PDA. (C) Double balloon exclusion of the proximal SMA after coil plus glue embolization of the inferior PDA showing no contrast opacification of the inferior PDA. Black arrow points to the embolization coil; white arrow points to the radiopaque glue cast in the inferior PDA. (D) DSA performed two weeks after the initial procedure shows the persistently occluded inferior PDA.

Pre-procedural computed tomography (CT) angiography revealed targets in both the SMA and celiac artery; the SMA was deemed the better target based on the extent of tumor contact (Fig. 1A). Following diagnostic angiography, an attempt was made to perform TAMP using right groin access. Following intravenous administration of 5000 units heparin, a RenovoCath catheter was advanced over a 0.014 microwire and positioned in the proximal SMA. Following inflation of the proximal and distal balloons, angiography was performed via the RenovoCath infusion port in the excluded SMA segment. The angiogram revealed an inferior PDA arising from the excluded SMA segment, precluding TAMP. Despite multiple manipulations of the RenovoCath catheter, including using the minimal distance between balloons (15 mm), the proximal PDA branch could not be excluded. The decision was made to embolize the PDA side branch and, if successful, proceed with TAMP. Embolizing a few arterial branches of either the SMA or the celiac artery is safe due to the extensive collateral arterial supply in the upper gastrointestinal tract.

To initiate the procedure, a 5 Fr Kumpe catheter was used to select the orifice of the inferior PDA; digital subtraction angiography (DSA) was performed to confirm appropriate positioning (Figs. 1A and B). A 2.4 Fr microcatheter was advanced through the Kumpe catheter into the inferior PDA and three 3/2 Tornado embolization coils (Cook Medical, Bloomington, IN) were deployed. A repeat angiogram demonstrated persistent albeit diminished flow across the coil pack. To achieve complete occlusion enabling TAMP, additional glue embolization of the inferior PDA was performed using N-butyl cyanoacrylate mixed 1:1 with lipiodol. Post-embolization DSA of the proximal SMA showed the absence of flow in the inferior PDA (Fig. 1C) which was confirmed to be maintained after the second TAMP treatment 2 weeks later (Fig. 1D). The RenovoCath was then positioned back into the proximal SMA. The catheter balloons were inflated, and angiography was performed in the excluded SMA segment. The inferior PDA was noted to be nonopacified with the contrast dwelling between the inflated balloons without proximal or distal escape. The injector was connected to the RenovoCath, and 120 mL of gemcitabine solution was infused over 20 minutes at a rate of 6 mL/minute, successfully completing TAMP treatment.

**Fig. 2:**
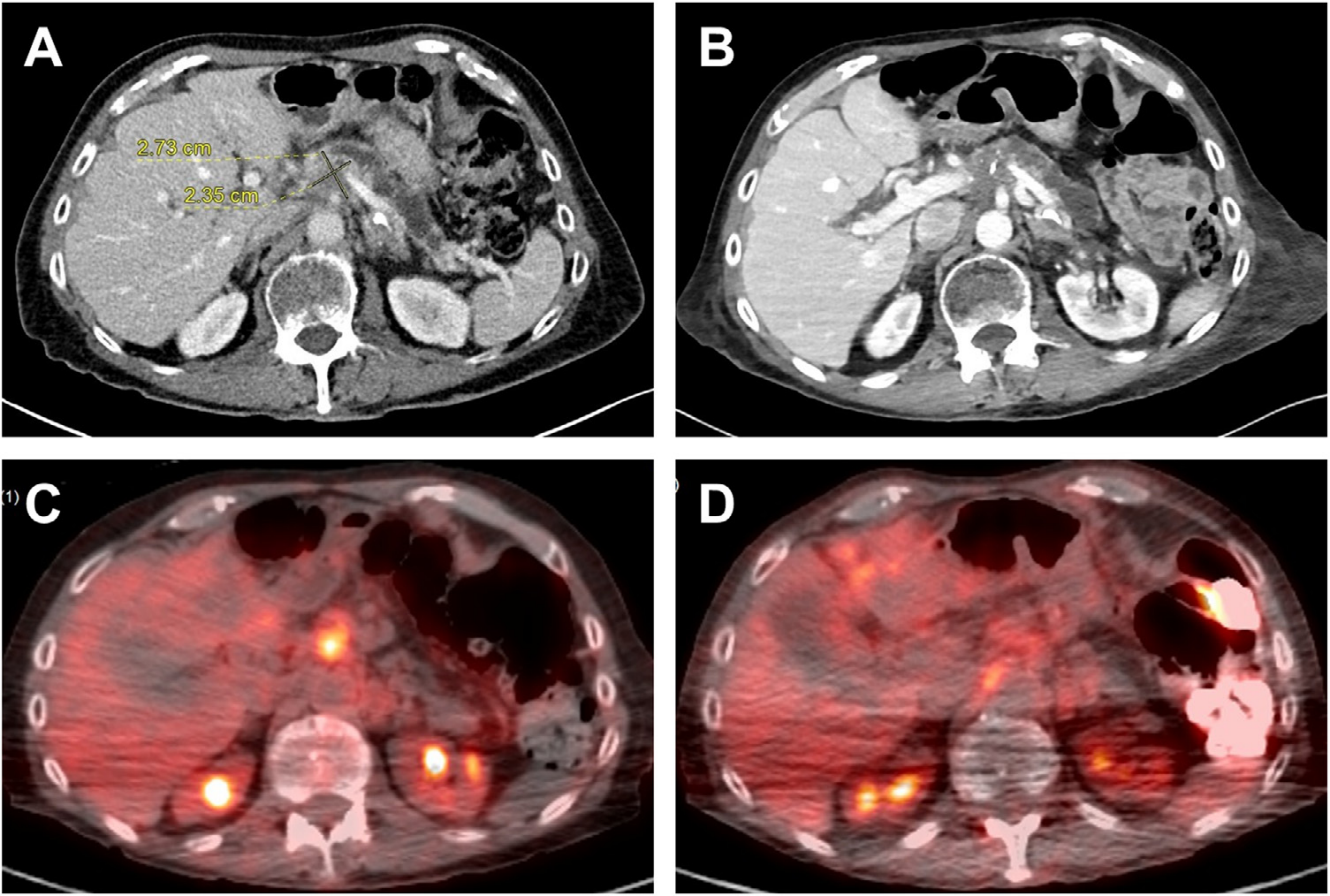
Computed tomography (CT) and positron emission tomography-computed tomography (PET-CT) images of the abdomen at the level of the pancreatic tumor. A-B: Images from CT scans performed before the initial TAMP treatment (A) and after the eighth TAMP treatment (B). C-D: Images from PET-CT scans performed before the initial TAMP treatment (pancreatic tumor standardized uptake value [SUV] 6.4) (C) and after the eighth TAMP treatment (SUV 3.1) (D).

The patient tolerated all 8 TAMP procedures (twice per month) without complications. Post eighth treatment at 4-months follow-up, CT scans revealed stable disease relative to scans performed prior to the first TAMP treatment (Figs. 2A and B), whereas positron emission tomography-computed tomography (PET-CT) revealed a 52% reduction in fluorodeoxyglucose activity at the site of treatment (Figs. 2C and D).

## Discussion

To our knowledge, this was the first successful attempt to embolize an arterial side branch and perform TAMP in the same setting. In our ongoing clinical trial utilizing TAMP for targeted gemcitabine delivery in patients with LAPC, very few coil embolization procedures have been performed, and in all cases TAMP could not be performed in the same setting as the embolization procedure due to leakage through the coil pack. Using liquid embolic alone in a jejunal or duodenal branch involves the risk of distal embolization and focal bowel ischemia with ulceration [3]. In our case, the combination of coil plus glue achieved the goal of complete embolization without risk of bowel ischemia as the coil pack prevented distal glue migration while the glue plug withstood the elevated perfusion pressure during TAMP. Furthermore, the embolization approach utilized was stable, safely enabling the patient to receive the full course of 8 TAMP-gemcitabine treatments. In cases where arterial anatomy may otherwise preclude the use of TAMP, coil plus glue embolization of side branches may successfully prevent immediate washout and enable the intra-arterial pressure required to drive the therapeutic across the vessel wall for localized targeted treatment. In an ongoing Phase 3 trial (TIGeR-PaC, NCT03257033) of patients with locally advanced pancreatic cancer, localized gemcitabine delivery via TAMP is proving to significantly reduce systemic side effects [4]. However, the inability to exclude side branches can preclude the use of intra-arterial pressure mediated TAMP. The RenovoCath catheter was approved by the Federal Drug Administration in 2014 to be used in any peripheral vascular bed for delivery of chemotherapy. Therefore, the utilization of this catheter to treat tumors in different parts of the body is expected to increase. The embolization approach outlined in our case report may provide a viable and safe option for enabling TAMP in situations of challenging arterial anatomy.

## Conclusion

Vessel side branch exclusion, which can be difficult with challenging arterial anatomy, is a prerequisite for achieving the intra-arterial pressure that drives the infusate across the vessel wall during TAMP. In cases where arterial anatomy may otherwise preclude TAMP, coil plus glue embolization of side branches may provide a potentially viable and safe solution in carefully selected cases.

## Data availability

All data generated or analyzed during this study are included in the submitted manuscript.

## Patient consent

A written informed consent was obtained from the patient for the publication of this case report.
